# A High‐Throughput Drug Repurposing Strategy to Treat TBX2 and/or TBX3 Dependent Cancers

**DOI:** 10.1002/cam4.70303

**Published:** 2024-10-15

**Authors:** Jenna S. Bleloch, Sizhu Lu, Saif Feroz Khan, Karabo Serala, Elena Seraia, Val Millar, Daniel Ebner, Colin Goding, Sharon Prince

**Affiliations:** ^1^ Division of Cell Biology, Department of Human Biology, Faculty of Health Sciences University of Cape Town, Observatory Cape Town South Africa; ^2^ Ludwig Institute for Cancer Research, Nuffield Department of Clinical Medicine University of Oxford Oxford UK; ^3^ Target Discovery Institute, Nuffield Department of Medicine University of Oxford Oxford UK

**Keywords:** drug repurposing, high‐throughput screen, T‐box factors, transcription factors

## Abstract

**Background:**

The highly homologous T‐box transcription factors TBX2 and TBX3 are critical for embryonic development, and their overexpression in postnatal tissues contributes to a wide range of malignancies, including melanoma and rhabdomyosarcoma. Importantly, when TBX2 and TBX3 are depleted in cancers where they are overexpressed, the malignant phenotype is inhibited, and they have therefore been regarded as druggable targets. However, the time and costs associated with de novo drug development are challenging and result in drugs that are costly, especially for patients in low‐ and middle‐income countries. In the current study, we therefore combined a targeted and drug repurposing approach to identify drugs that are expected to be more efficacious and cost‐effective with significantly reduced side effects.

**Methods:**

A high‐throughput cell‐based immunofluorescence screen was performed to identify drugs in the Pharmakon 1600 drug library that can negatively regulate TBX2 and/or TBX3 levels. “Hit” drugs were validated for their effect on TBX2/TBX3 levels and cytotoxicity in TBX2/TBX3‐dependent melanoma and rhabdomyosarcoma cells. To this end, immunofluorescence, western blotting, quantitative real‐time PCR, and MTT cell viability assays were performed.

**Results:**

Niclosamide, piroctone olamine, and pyrvinium pamoate, were identified as TBX2 and/or TBX3‐targeting drugs, and they exhibited cytotoxicity in a TBX2/TBX3‐dependent manner. Furthermore, these “Hit” drugs were shown to induce senescence and/or apoptosis.

**Conclusions:**

Niclosamide, piroctone olamine, and pyrvinium pamoate are promising, cost‐effective therapeutic agents for the treatment of TBX2/TBX3‐dependent cancers.

## 1 Introduction

The elucidation of the complex molecular mechanisms underpinning cancer has been critical for identifying central regulators of the disease that can be targeted in the development of highly specific and effective treatments with negligible adverse effects [1]. Importantly, many oncogenic signalling pathways converge on transcription factors which are key mediators of cancer development and progression, and they therefore represent attractive therapeutic targets [2]. Examples include the highly homologous developmental regulators TBX2 and TBX3, which are members of the conserved T‐box family of transcription factors.

TBX2 and TBX3 are dynamically expressed in many tissues and organs during embryonic development, where they play highly specific and critical roles in processes ranging from the specification of the primary germ layers to limb patterning and organogenesis [3, 4, 5]. They have no known functions in post‐natal tissues [6, 7, 8] but TBX2 and TBX3 are frequently overexpressed in melanoma, carcinomas and sarcomas, where they contribute to several oncogenic processes. These include the bypass of senescence and apoptosis, promotion of proliferation, tumour formation, invasion, and migration, and conferring tumour drug resistance [9, 10]. Interestingly, while highly homologous, in cancers where they are both overexpressed, such as melanoma, TBX2 and TBX3 have central but distinct roles where TBX2 functions as a potent growth promoting factor and TBX3 contributes to tumour formation and invasion [9, 10]. Furthermore, there is strong in vitro and in vivo biological evidence that TBX2 and TBX3 are novel targets for the development of anti‐cancer drugs that can be administered on their own or in combination with current chemotherapeutic DNA‐damaging drugs such as cisplatin [11, 12, 13, 14, 15, 16]. However, the pipeline to develop de novo drugs, especially those that target oncogenic factors, is long, arduous, and expensive, and transcription factors themselves have often been viewed as undruggable owing to the absence of any intrinsic catalytic activity. However, it is possible that drugs targeting expression, stability or activity might prove effective in modulating transcription factor function. This study therefore explored targeting TBX2/TBX3 with commercially available non‐cancer drugs. The advantage of this strategy is that it combines a targeted approach, which is expected to have significantly reduced side‐effects, with a repurposing approach which will facilitate cost‐effective drug development as it circumvents many steps along the drug development pipeline. Indeed, drug repurposing exploits the fact that several diseases are underpinned by common molecular pathways and can therefore be treated with the same drugs [17]. Importantly, since the drugs are already approved for clinical use for other purposes, it reduces the time and costs of drug development, and they can potentially be applied immediately to impact the lives of cancer patients [18]. This is especially important in low‐ and middle‐income countries (LMICs) where new therapies do not readily translate to health benefits due to their associated costs [19].

In this study, we conducted a high‐throughput cell‐based immunofluorescence screen to identify commercially available drugs that negatively regulate TBX2 and/or TBX3 levels in TBX2/TBX3‐driven melanoma cells and identify several that exhibit anti‐cancer activity in melanoma and rhabdomyosarcoma by inhibiting TBX2 and/or TBX3. Specifically, we provide evidence that the anthelmintics niclosamide and pyrvinium pamoate, and the anti‐fungal agent piroctone olamine may have the potential to be repurposed for the treatment of TBX2/TBX3‐driven cancers either as single agents or in combination with currently used chemotherapeutics.

## 2 Materials and Methods

### 2.1 Cell Culture

501mel human melanoma and RH30 human alveolar rhabdomyosarcoma (ARMS) cells (kindly provided by Associate Professor Judith Davie, Southern Illinois University) were cultured in Roswell Park Memorial Institute Medium (RPMI)‐1640 (Sigma Aldrich, Missouri, USA). RD human embryonal rhabdomyosarcoma (ERMS) cells (ATCC CCL‐136) were cultured in Dulbecco's Modified Eagle's Medium (DMEM) (Sigma Aldrich). All culture medium was supplemented with 10% heat‐inactivated foetal bovine serum (FBS), 100 U/mL penicillin and 100 μg/mL streptomycin. Cells were maintained at 37°C in a 95% air and 5% CO_2_ humidified incubator. Medium was replaced every 2–3 days, and cells were routinely subjected to mycoplasma tests. Only mycoplasma‐free cells were used in experiments.

### 2.2 Inducible FLAG‐TBX2/3501mel Cells and CRISPR/Cas9‐Induced Pluripotent Stem Cells (iPSCs)

Monoclonal inducible stable 501mel cell lines expressing 3XFLAG‐tagged TBX2 and 3XFLAG‐tagged TBX3 were previously established using a tetracycline‐controlled transcriptional activation (Tet‐On) system combined with the piggyBAC (PB) transposase‐mediated genomic integration system [20]. Inducible FLAG‐TBX2 501mel monoclonal cell line clone 8 and 501mel inducible FLAG‐TBX3 monoclonal cell line clone 9 were selected to be used in this study. iPSCs with a Tet‐On CRISPR/Cas9 system (established and kindly provided for use by Professor Musa Mhlanga, Radbound Universiteit, Netherlands) were cultured in feeder‐free StemPro iPSC medium which included DMEM/F12, GlutaMAX supplement (10565042) supplemented with 2% StemPro hESC SFM (A1000701), 1.8% bovine serum albumin (BSA) (A1000801), 55 mM 2‐mercaptoethanol (21985023) and 10 μg/mL FGF‐Basic (AA 1–155) Recombinant Human Protein (PHG0264) all from Thermo Fisher (Massachusetts, USA).

### 2.3 Small Molecule Library Screen

Inducible FLAG‐TBX2 501mel cells were harvested, counted, and resuspended in FBS‐free medium at 1 × 10^6^ cells/mL. CellTracker Orange (34551, Thermo Fisher Scientific, Massachusetts, USA) (1:1000) was added, and cells were incubated for 45 min at 37°C, spun and resuspended in 1 mL FBS‐free medium and incubated for another 30 min at 37°C. Cells were spun, resuspended in normal medium, and counted. A 1:1 mixture of stained inducible FLAG‐TBX2 501mel cells and inducible FLAG‐TBX3 501mel cells was prepared at 10,000 cells/mL final, and 80 μL (~400 from each population) were seeded in 30 x 384‐well clear‐bottom Greiner μClear plates (781,097, Greiner Bio‐One, Austria) using a FlexDrop PLUS cell dispenser (PerkinElmer, Massachusetts, USA), which was then placed into a cell culture incubator. From this point, all liquid handling steps were performed using a Perkin Elmer JANUS Automated Workstation equipped with a 384‐well head (PerkinElmer, Massachusetts, USA) unless otherwise stated. Once the cells adhered, medium was removed and fresh medium containing 20 ng/mL doxycycline was added (75 μL per well), and cells were incubated for 24 h. The Pharmakon 1600 drug library (5 library plates) was predispensed (640 nL of 10 mM drug in dimethyl sulfoxide (DMSO) (D8418; Sigma Aldrich, Missouri, USA)) into 384‐well round bottom intermediate plates (Corning 3656, Scientific Laboratory Supplies) using a LABCYTE Echo 550 (Agilent Technologies, California, USA). in the drug was diluted with 40 μL medium to give 160 μM (6.25 x concentration) and 5 μL added to each cell culture assay plate (6 assay plates per library plate (3 time points in duplicate)) to achieve a final concentration of 10 μM. After 4 h, 12 h and 24 h medium was removed, and wells were washed thrice with PBS, followed by fixation with 4% paraformaldehyde for 15 min at RT after which plates were washed 3x with PBS again and stored at 4°C until immunofluorescence processing. Cells were blocked and permeabilised with PBS containing 5% BSA and 0.2% Triton‐X‐100 for 20 min at RT and then incubated with mouse monoclonal anti‐FLAG M2 (1:500) (Sigma Aldrich, Missouri, USA) for 1 h at RT in blocking buffer. Wells were washed 3x with PBS and incubated with Alexa Fluor 488 secondary antibody and DAPI (1:1000) for 1 h at RT in the dark. Wells were washed 3x with PBS and imaged with an IN Cell Analyser 6000 (GE Healthcare Life Sciences, Illinois, USA) using the 20x 0.75 NA air objective, on line cell counting to 1000 cells/well (up to 16 fields of view). And the following channel settings: UV DAPI: 75 ms, confocal 0.99 AU; FITC: 2500 ms, confocal 1.23 AU; and dsRED: 100 ms, open aperture.

### 2.4 Automated Parameter Extraction From Images

A customised parameter extraction protocol was developed using IN Cell Developer Toolbox v1.6.2 (GE Healthcare Life Sciences, Illinois, USA) for automated image analyses. Briefly, this protocol segmented nuclei of cells (UV channel), separated the FLAG‐TBX2 (CellTracker Orange stained cells) and FLAG‐TBX3 cell populations (dsRED channel) for independent analyses and measured whole cell, nuclear, cytoplasmic, eroded nuclear and nuclear collar FITC fluorescence intensity (FITC channel).

### 2.5 Z‐Score ‘Hit’ Identification

Mean nuclear, eroded nuclear and nuclear collar FITC fluorescence intensity measurements representing TBX2/3 protein levels in defined areas of the cell were used for analyses to identify ‘hit’ compounds. Mean nuclear FITC fluorescence was used as the end‐point parameter for total protein levels as TBX2/3 localises to the nucleus, and the end‐point parameter used to indicate subcellular localisation was calculated as follows:

$$\text{Nuclear}/\text{cytoplasmic ratio}=\frac{\text{Mean eroded nuclear FITC fluorescence}}{\text{Mean nuclear collar FITC fluorescence}}$$

A mean nuclear/cytoplasmic ratio > 1 indicates nuclear localisation, and a ratio < 1 indicates cytoplasmic localisation. The parameter for total protein levels and the parameter for subcellular localisation for each drug treated well were normalised to their respective averaged vehicle control parameter. This was done on a plate‐to‐plate basis to account for plate‐to‐plate variation in assay performance. The normalised parameter measurements for the technical repeats were averaged as an *R* ^2^ correlation > 0.8 and a Pearson's correlation > 0.9 were calculated for each duplicate. To identify ‘hit’ compounds a *z*‐score was calculated for each normalised parameter for each drug treatment. A *z*‐score assumes that the data are normally distributed and is a measure of the number of standard deviations below or above the population mean that a data point is and was calculated as follows:

$$z-{\text{score}}_{i}=\frac{\left(S_{i}-\text{mean}\left(S_{\text{plate}}\right)\right)}{\mathrm{Std}\left(S_{\text{plate}}\right)}$$

*z*‐score_i_ is the *z*‐score determined for the *i*th sample ($S_{i}$) by dividing the difference between $S_{i}$ and the sample mean of the plate ($S_{\text{plate}}$) by the standard deviation of the plate. *z*‐scores ≥ 2 or ≤ 2 (i.e., 2 standard deviations above or below the sample mean) are considered ‘hits’ that are worth taking forward for validation [21, 22]. These ‘hits’ were filtered by a cell count threshold of at least 25 cells/FOV. Our study aimed to identify FDA‐approved drugs that negatively regulate TBX2 and/or TBX3 (i.e., reduced nuclear fluorescence levels and nuclear/cytoplasmic ratios of < 1) and thus we were interested in *z*‐scores ≤ 2 (i.e., 2 standard deviations below the sample mean).

### 2.6 Chemical Compounds and Small Molecule Library Treatments

Doxycycline (D9891, Sigma Aldrich, Missouri, USA) was used (20 ng/mL–200 ng/mL) to induce expression of inducible FLAG‐TBX2 and inducible FLAG‐TBX3 501mel cell lines. The Pharmakon 1600 drug library (MicroSource Discovery Systems, Connecticut, USA) contains 1600 known drugs (10 mM in DMSO) from US and International Pharma. All compounds within the Pharmakon 1600 collection have reached clinical evaluation and demonstrate biological activity against known targets. Cells were treated with the library at a concentration of 10 μM. The following small compounds were purchased for further validation and characterisation: niclosamide (N3510), tacrolimus (Y0001933), piroctone olamine (51872), pyrvinium pamoate (P0027) (Sigma Aldrich, Missouri, USA), and vardenafil hydrochloride (sc‐220,368, Santa Cruz Biotechnology, Texas, USA). These drugs were dissolved in DMSO to give a 5 mM stock which was stored at −20°C.

### 2.7 Cell Viability Assays

Cells were seeded in 96‐well plates and treated the next day at a confluency of 60%. For niclosamide and pyrvinium pamoate cell viability assays, the cells were treated for 72 h with 2, 4, 6, 8, 10 μM of drug and 10 μM vehicle (DMSO) and for piroctone olamine cells were treated for 72 h with 4 μM, 8 μM, 12 μM, 16 μM, 20 μM of drug and 20 μM vehicle (DMSO). Cell viability was measured using the 3‐(4,5‐dimethylthiazol‐2‐yl)‐2,5‐diphenyl‐trazolium bromide (MTT) assay (M2128, Sigma Aldrich, Missouri, USA) according to the manufacturer's instructions. Mean cell viability for each treatment condition was calculated as a percentage of the mean vehicle control. At least three independent experiments in quadruplicate were performed from which the half maximal effective concentration (EC_50_) was determined using GraphPad Prism version 6.0 (GraphPad Software, California, USA).

### 2.8 Western Blot Analyses

Cells were harvested and proteins prepared as described previously [23]. The following primary antibodies, diluted 1:1000 unless otherwise stated, were used in this study: goat polyclonal antibody to TBX2 (sc‐17880) from Santa Cruz Biotechnology (Texas, USA); mouse monoclonal antibody to FLAG M2 (F1804) and rabbit polyclonal antibody to TBX3 (ab99302) (Abcam, UK); PARP (#9542) from Cell Signalling Technology and Flag (F1804) from Sigma Aldrich (Missouri, USA). Densitometry readings were obtained using ImageJ v1.50i [24] and protein expression levels were represented as a ratio of protein of interest/p38 loading control normalised to the vehicle treated control sample if appropriate. All blots are representative of at least two independent repeats.

### 2.9 Immunofluorescence

Immunofluorescence was performed as previously described [25]. Mouse monoclonal antibody to FLAG M2 (F1804) (1:500) (Sigma Aldrich, Missouri, USA) was used in this study. After PBS washes, coverslips were incubated with the appropriate secondary antibody diluted 1:1000 in blocking buffer for 1 h at RT in the dark. The secondary antibodies used included donkey anti‐rabbit Cy3, donkey anti‐rabbit, donkey anti‐mouse and donkey anti‐goat Alexa Fluor 488 (Jackson ImmunoResearch Laboratories Inc. Pennsylvania, USA or Thermo Fisher Scientific, Massachusetts, USA). A secondary antibody‐only control was always included and used to eliminate background signal when imaging. Nuclei of cells were stained with Hoechst (33,342, Invitrogen, California, USA) or 4′,6‐diamidino‐2‐phenylindole (DAPI) (Thermo Fisher Scientific, Massachusetts, USA) either simultaneously with secondary antibody incubation (1:1000) or afterwards for 10 min at RT in the dark. After 1 X PBS washes, coverslips were mounted using Mowiol mounting medium. Cells were imaged with an LSM 510, 710 or 880 confocal microscopes (Zeiss, Germany) using a Plan‐Apochromat 63x/1.40 Oil DIC objective. In most cases, multiple *z* layers were acquired with 1 μm step width, images were processed using ZEN 2012 imaging software (Zeiss, Germany) and maximum intensity projections were generated. For quantification, mean fluorescence was measured from at least 20 fields of view (FOV) per treatment condition and pooled from three independent repeats.

### 2.10 Proof of Concept: TBX2/3 Knockdown Using siRNAs

501mel, RD and RH30 cells were seeded in 96‐well and 6‐well plates and treated the following day at 60% confluency with EC_50_ concentrations of niclosamide, piroctone olamine, pyrvinium pamoate or vehicle for 72 h. After 24 h of drug treatment, 20 nM of siControl (non‐silencing, siCTRL, 1027310, Qiagen, USA) siTBX2 (SI03238802, Qiagen, USA) or siTBX3 (SI03100426, Qiagen, USA) was added to the drug‐containing medium of the cells to transiently knockdown TBX2 or TBX3 respectively and 48 h later MTT assays (96‐well plate) as described earlier.

### 2.11 Quantitative Real‐Time PCR (qRT‐PCR)

Inducible CRISPR/Cas9 iPSCs were seeded in a 24‐well plate and treated 3 days later at 60% confluency with 20 ng/mL doxycycline for 24 h to induce Cas9 expression. Niclosamide, piroctone olamine, pyrvinium pamoate, tacrolimus and vehicle were added to the medium of the cells to achieve a final concentration of 10 μM and left for 24 h after which total RNA was extracted using Direct‐zol RNA MiniPrep Plus (Zymo Research, California, USA), reverse transcribed using the ImProm‐II Reverse Transcription System (A3800, Promega, Wisconsin, USA) and qRT‐PCR was conducted with the KAPA SYBR FAST qPCR Master Mix (2X) Kit (KR0389, Massachusetts, USA) according to the manufacturer's instructions. Primers specific to Cas9 (Forward‐5′‐GCGATCAGATTTTCCAGCCG‐3′, Reverse‐5′‐ACATGATCAAGTTCCGGGGC‐3′) were kindly provided by Professor Musa Mhlanga, Radbound Universiteit, Netherlands and primers specific to β‐actin (F:‐5′‐CGGCATCGTCACCAACTG‐3′, R: 5’‐AAC ATGATCTGGGTCATCTTCTC‐3′), human TBX3 (F: CAATTCTCGGTGGATGGTG; R: GGCTGGTATTTGTGCATGG) were custom designed (Integrated DNA Technologies, Iowa, USA) and TBX2 (QT00091266; Qiagen, USA) and GUSB (QT00046046; Qiagen, USA) were ordered. LightCycler 3.0 (Roche, Switzerland) PCR cycle parameters were: denaturation for 3 min at 95°C, annealing and amplification for 40 cycles for 10 s at 95°C, 20 s at 55°C and 1 s at 72°C, melting for 15 s at 65°C and cooling for 30 s at 40°C. Each DNA sample was quantified in duplicate and a negative control without cDNA template was run with every assay to assess the overall specificity and monitor for contamination. Melting curve analyses were carried out to ensure product specificity, and data were analysed using the 2^−∆∆Ct^ method. Relative Cas9 mRNA expression levels were normalised to β‐actin expression levels, and data were represented as a fold change of Cas9 mRNA expression in Tet‐On CRISPR/Cas9 iPSCs induced with doxycycline and treated with drug or vehicle relative to uninduced and untreated Tet‐On CRISPR/Cas9 iPSCs.

### 2.12 Senescence‐Associated β‐Galactosidase Assay

Treated and control cells were seeded at 5000 cells/cm^2^ in 6‐well plates and were stained after 72 h of drug treatment using the Senescence β‐Galactosidase Staining Kit (#9860, Cell Signalling Technology, USA) following the manufacturer's protocol. Briefly, cells were washed once with 1 X PBS and fixed by adding 1 mL of 1 X fixative solution to each well for 15 min at RT. Subsequently, cells were washed with 1 X PBS and 1 mL of the β‐Galactosidase staining solution (pH = 6.0) was added to each well. The plate was sealed with parafilm (Fisher Scientific, USA) to prevent evaporation, and plates were incubated at 37°C O/N in a dry incubator (no CO_2_). The next day, images were taken using an EVOS M5000 Imaging System microscope (Thermo Fisher Scientific, USA).

### 2.13 Statistical Analyses

All data were obtained from at least three independent experiments (unless otherwise stated) with error bars representing standard error of the mean (SEM). Data were analysed using GraphPad Prism version 6.0 (GraphPad Software), and a parametric unpaired t‐test was performed. Significance was accepted at *p < 0.05, **p < 0.01 and ***p < 0.001.

## 3 Results

### 3.1 High‐Throughput Screen: Cell Culture Model and Experimental Design

**FIGURE 1:**
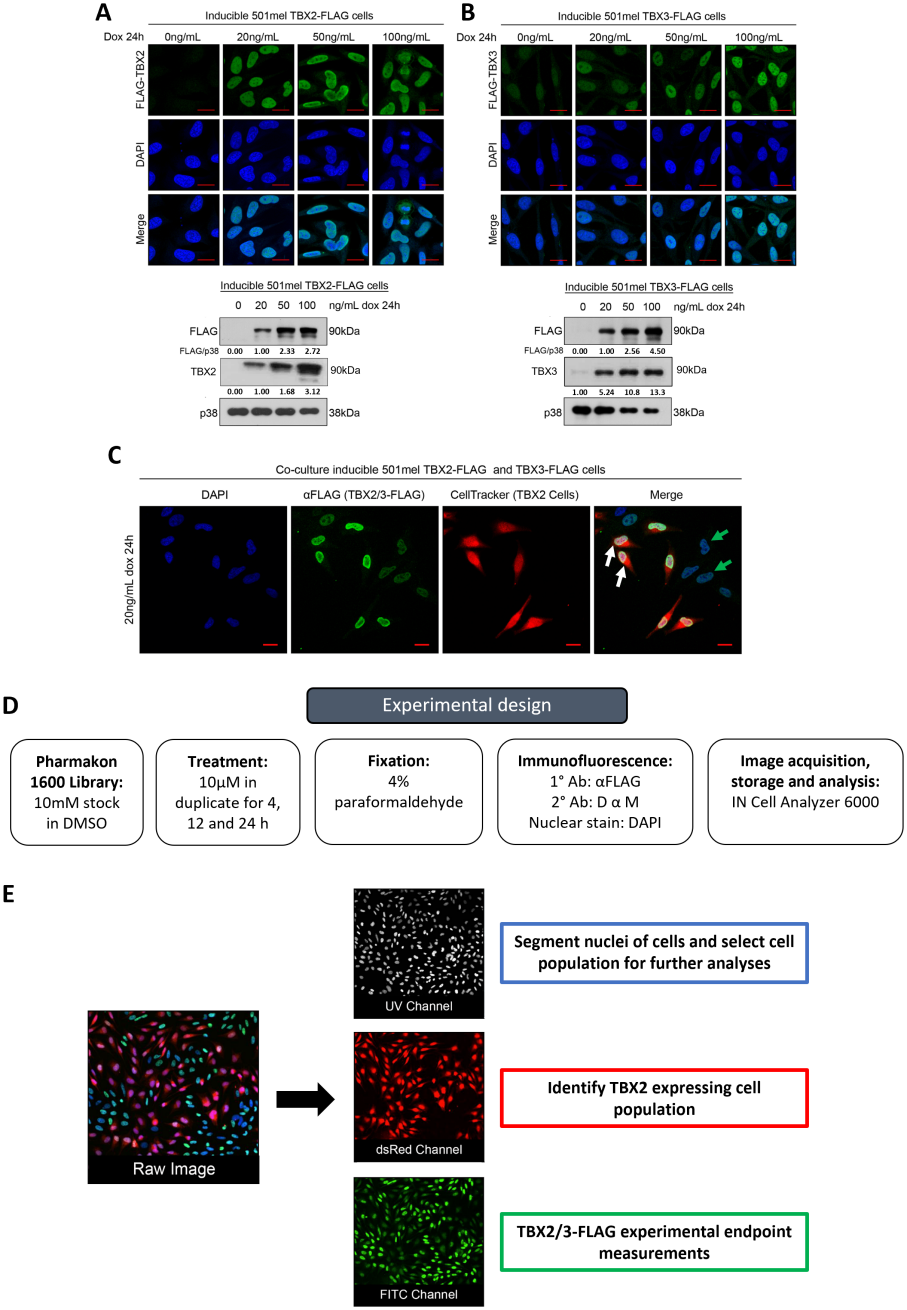
Tet‐On inducible cell culture model used in the high‐throughput screen. 501mel cell lines expressing an inducible FLAG‐tagged TBX2 and TBX3 treated with a range of doxycycline (dox) concentrations. Representative confocal immunofluorescence maximum intensity projection images (630X; Carl Zeiss LSM 880) and western blotting of (A) FLAG‐TBX2 and (B) FLAG‐TBX3. For immunofluorescence a FLAG primary antibody and Alexa Fluor 488 secondary antibody was used, and nuclei were stained with DAPI (scale bar is 20 μm) and for western blotting, antibodies used were as indicated and p38 was included as a loading control. Densitometry readings were obtained using ImageJ and protein expression levels are represented as a ratio of protein of interest/p38 normalised to the vehicle control sample. (C) Representative confocal immunofluorescence images (200X, 2X zoom; Carl Zeiss LSM 710) of co‐culture of FLAG‐TBX2 cells pre‐stained with CellTracker (DeepRed) and FLAG‐TBX3 cells treated with 20 ng/mL dox for 24 h and processed for immunofluorescence as described above. (D) Schematic of high‐throughput screen experimental design. (E) IN Cell Developer Toolbox customised parameter extraction protocol.

To identify drugs that downregulate TBX2/TBX3 levels and/or block their nuclear localisation and thus prevent them from regulating their target genes, we performed a high‐throughput screen that employed immunofluorescence as readout. Due to a lack of reliable and high‐grade antibodies against TBX2 and TBX3 for immunofluorescence, this study used a tetracycline‐on (Tet‐On) system that enabled the inducible expression of 3XFLAG‐tagged TBX2 or 3XFLAG‐tagged TBX3 in the 501mel human melanoma cell line. An advantage of this system is that it enables control of the levels of TBX2/TBX3 depending on the concentration of doxycycline, a tetracycline derivative, and thus avoids pleiotropic effects. In addition, we had previously confirmed key oncogenic or pro‐metastatic roles for TBX2 and TBX3 in 501mel cells [26, 27] and it was anticipated that any drug that impacts TBX2/TBX3 levels and nuclear localisation in these cells would do the same in other TBX2/TBX3‐driven cancers such as rhabdomyosarcoma (RMS). Immunofluorescence and western blotting show the nuclear localisation and dose dependent expression respectively of FLAG‐TBX2 (Figure 1A) and FLAG‐TBX3 (Figure 1B) after treatment of cells with doxycycline. Importantly, the immunofluorescence reveals that all cells that stained positively with the FLAG antibody exhibited nuclear localisation of TBX2 and TBX3 while the western blots show that there was a dose‐dependent increase in FLAG‐TBX2/FLAG‐TBX3 levels. The lowest concentration of doxycycline able to induce detectable levels of FLAG‐TBX2/TBX3 was 20 ng/mL and, this concentration was used for all further experiments. To reduce the time and cost of conducting two separate screens, a FLAG‐TBX2 expressing cell population was pre‐stained with CellTracker and co‐cultured with a FLAG‐TBX3 cell population which enabled the FLAG‐TBX2 and FLAG‐TBX3 cell populations to be distinguished from one another in a single screen (Figure 1C). The co‐cultures were treated with each drug in the MicroSource Pharmakon 1600 library or DMSO, which was included as a vehicle control (Figure 1D).

This library comprises a collection of drugs that have all reached clinical evaluation with most still in the market and was chosen as it potentially enables repurposing of drugs with known pharmacokinetics and safety profiles. After drug treatment, cells were processed for immunofluorescence, imaged, and data stored and analysed on an IN Cell Analyser 6000.

### 3.2 High‐Throughput Screen: Image Analyses for Parameter Extraction

The IN Cell Analyser 6000 captured a composite image derived from three channels for each field of view (Figure 1E). The UV channel was used to identify DAPI stained nuclei and determined the population of cells that were taken for further analyses. Furthermore, the UV channel provided additional measurements which include cell count, nuclear area, nuclear form factor and length/width ratio, nuclear integrity as measured by fluorescence intensity and its standard deviation (Table 1). The dsRed channel was used to detect the CellTracker Orange stained FLAG‐TBX2 cell population (Figure 1E), and the FLAG‐TBX2 and FLAG‐TBX3 cell populations were then analysed independently for their FLAG expression in the FITC channel (Figure 1E). Nuclear FITC fluorescence was measured using the DAPI mask and cytoplasmic FITC fluorescence was determined by subtracting nuclear fluorescence from whole cell fluorescence. To determine subcellular localisation, nuclear FITC fluorescence was divided by cytoplasmic FITC fluorescence where a ratio of > 1 indicates more nuclear localisation and < 1 indicates more cytoplasmic localisation. The latter was also determined by measuring an eroded FITC nucleus which represents nuclear expression and a FITC nuclear collar which represents cytoplasmic expression followed by the same ratio calculation. Extracted and calculated parameters are described in Table 1.

**TABLE 1:** Details of extracted and calculated parameters.

Parameters	Description
CountDividedbyFOV	Online cell counting to 1000 cells or maximum of 16 field of views (FOVs) imaged per well. Nuclei count (DAPI)/FOV gives an indication of cell number
AreaNuc	Area of nucleus from DAPI stain
MeanIntNuc	Mean DAPI intensity in the nucleus
DxANuc	AreaNuc multiplied by MeanIntNuc
FormFactorNuc	Nucleus shape scored by roundness (DAPI stain). Round = 1 which indicates cell death
NuclearAreaFactor	Nuclear area factor is AreaNuc/FormFactorNuc. It is used as an early indicator of cell death
STDIntNuc	Standard deviation of nuclear intensity DAPI (MeanIntNuc) indicates if there is apoptosis that is, nuclear fragmentation
LengthWidthRatioNuc	Length/width ratio determined from DAPI stain
MeanInt FITCCell	Mean FITC (FLAG‐TBX2/TBX3) intensity of whole cell
MeanInt FITCNuc	Mean FITC (FLAG‐TBX2/TBX3) intensity of nucleus
MeanInt FITCCyto	Mean FITC (FLAG‐TBX2/TBX3) intensity of cytoplasm (MeanIntFITCNuc subtracted from Mean FITCCell)
RatioNucCytoFITC	Ratio of MeanIntFITCNuc divided by MeanIntFITCCyto. Indicates localisation of FLAG‐TBX2/TBX3: > 1 = nucleus and < 1 = cytoplasm
RatioNucCytoFITCCellsCollar	Ratio of mean erroded nuclear FITC divided by mean cytoplasmic collar FITC. Indicates localisation of FLAG‐TBX2/TBX3: > 1 = nucleus and < 1 = cytoplasm

### 3.3 Identification of TBX2 and TBX3 Targeting ‘Hit’ Drugs

‘Hit’ drugs were identified using a *z*‐score cut‐off of ≤ 2 and a cell count threshold of at least 25 cells per field of view [21, 22]. Briefly, *z*‐scores were calculated by measuring the number of standard deviations below or above the population mean for each of the following two experimental end‐point parameters: (1) downregulation of TBX2/3 levels represented by reduced whole cell FITC fluorescence intensity and (2) cytoplasmic localisation represented by nuclear/cytoplasmic FITC fluorescence intensity ratios of < 1. The cell count threshold was important because it enabled identification of drugs that could be further characterised for the mechanism by which they inhibit TBX2/3 levels and nuclear localisation.

**FIGURE 2:**
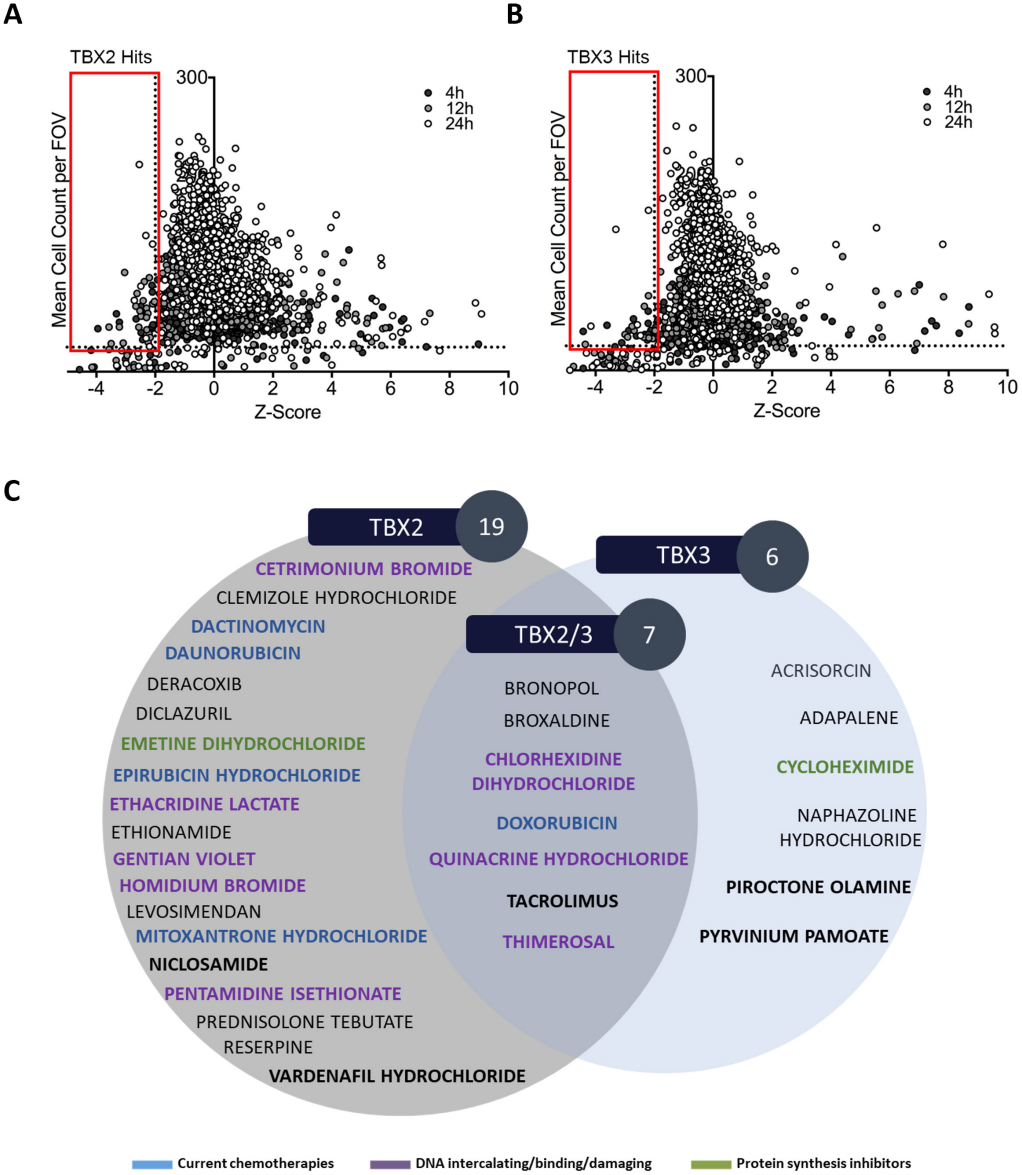
*z*‐score identification of commercially available ‘hit’ drugs that downregulate TBX2 and/or TBX3 protein levels. *z*‐score scatterplots of (A) FLAG‐TBX2 and (B) FLAG‐TBX3 expressing cells after 24 h of doxycycline induction (20 ng/mL) followed by Pharmakon drug library treatment (10 μM) for 4, 12 and 24 h. *z*‐scores were calculated from normalised mean FLAG‐TBX2/3 nuclear fluorescence data (*z* = (*x*‐μ)/σ, where *x* = well (drug) mean nuclear FLAG‐TBX2/3 fluorescence intensity value, μ = population (384‐well plate) mean FLAG‐TBX2/3 fluorescence intensity value and σ = population standard deviation). It was assumed that the data was normally distributed, and calculations were determined from two technical duplicates. A *z*‐score cut‐off of < −2 (i.e., more than 2 standard deviations below the population mean) and a cell count cut‐off of > 25 cells per FOV were used to select ‘hit’ compounds (red box on graphs) that downregulate TBX2 and or TBX3. (C) Venn diagram showing the ‘hit’ compounds identified in (A) and (B) above where 19, 6 and 7 compounds were identified to downregulate TBX2, TBX3 and both TBX2 and TBX3 total protein levels respectively. Compound names in blue represent already existing chemotherapeutics, purple represent DNA intercalating/binding and/or damaging agents and green are known protein synthesis inhibitors. Bolded compounds were taken further for validation.

**FIGURE 3:**
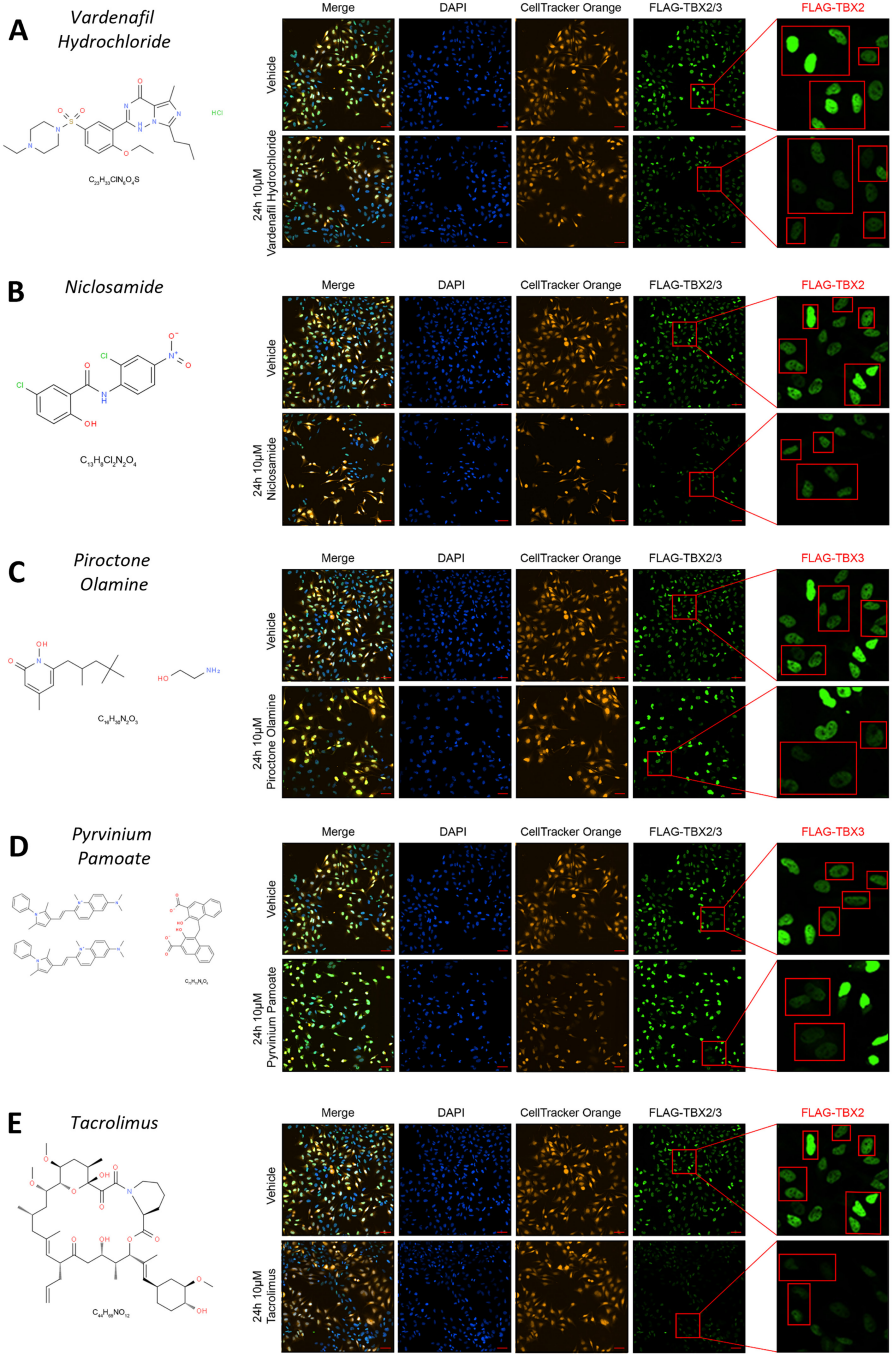
High‐throughput screen immunofluorescence images of selected ‘commercially available ‘hit’ drugs that downregulate TBX2 and TBX3 protein levels. Representative IN Cell Analyser 6000 images of inducible FLAG‐TBX2 501mel and inducible FLAG‐TBX3 501mel co‐cultured cells treated for 24 h with 20 ng/mL doxycycline followed by 10 μM (A) vardenafil hydrochloride (ChemSpider ID: 8318471) (B) niclosamide (ChemSpider ID: 4322) (C) piroctone olamine (ChemSpider ID: 45574) (D) tacrolimus (ChemSpider ID: 393220) (E) pyrvinium pamoate (ChemSpider ID: 10152945) or vehicle for 24 h. The FLAG‐TBX2 population was pre‐stained with CellTracker Orange and a FLAG antibody with an Alexa Fluor 488 conjugated secondary antibody were used to detect FLAG‐tagged TBX2 and TBX3. Nuclei were stained with DAPI. The scale bar is 60 μm. Close up images in the far‐right panel show (A, B, E) FLAG‐TBX2 and (C, D) FLAG‐TBX3 expressing cells in red outlined boxes with unboxed cells expressing (A, B, E) FLAG‐TBX3 and (C, D) FLAG‐TBX2 (identified from CellTracker Orange staining).

Figure 2A,B show scatter plots of ‘hits’ identified to downregulate total TBX2 and TBX3 levels respectively. According to the ‘hit’ selection criteria, 19 Pharmakon compounds were identified to significantly downregulate TBX2 levels, 6 to significantly downregulate TBX3 levels and 7 that significantly downregulated both TBX2 and TBX3 levels (Figure 2C). These included protein synthesis inhibitors (indicated in green) and DNA damaging agents (indicated in purple). In addition, several current chemotherapeutic agents (indicated in blue) such as epirubicin hydrochloride, dactinomycin, daunorubicin and doxorubicin were identified which suggests that anti‐cancer drugs currently in use may be functioning, in part, through targeting TBX2/3 levels (Livshits, Rao, and Smith 2014). The current study was interested in compounds that are not currently approved to treat cancer and based on preliminary literature searches the following ‘hits’ were selected for validation and characterisation: (i) the TBX2 ‘hits’, vardenafil hydrochloride and niclosamide (Figure 3A,B); (ii) the TBX3 ‘hits’, piroctone olamine and pyrvinium pamoate (Figure 3C,D); and (iii) the TBX2 and TBX3 ‘hit’, tacrolimus (Figure 3E).

### 3.4 Niclosamide, Piroctone Olamine and Pyrvinium Pamoate Are TBX2/3 Targeting Drugs

**FIGURE 4:**
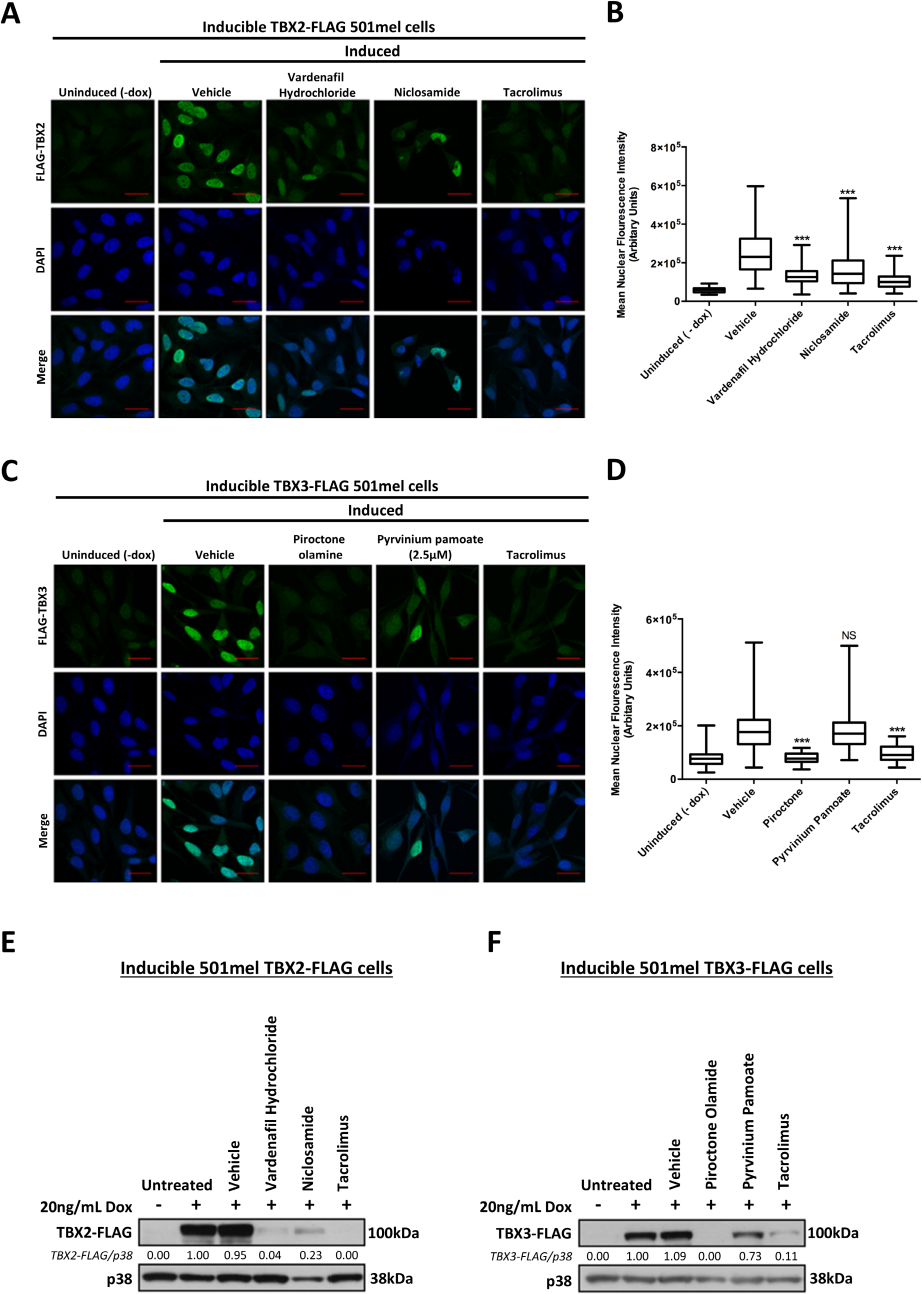
Validation of selected ‘hit’ compounds identified to downregulate TBX2 and TBX3 protein levels. (A, C). Representative confocal immunofluorescence maximum intensity projection images (630X; Carl Zeiss LSM 880) of inducible (A) FLAG‐TBX2 (C) FLAG‐TBX3 501mel cells induced with doxycycline (dox) for 24 h followed by 10 μM drug (2.5 μM for pyrvinium pamoate due to cell sensitivity) or vehicle treatment for 24 h. Ectopic FLAG‐TBX2 and FLAG‐TBX3 was detected with a FLAG antibody and an Alexa Fluor 488 conjugated secondary antibody. Nuclei of cells were stained with DAPI. Scale bar is 20 μm. (B, D) Box plots represent quantification of (B) FLAG‐TBX2 (D) FLAG‐TBX3 levels per treatment condition as mean nuclear Alexa Fluor 488 fluorescence from 20 fields of view pooled from three independent repeats. Data was analysed using GraphPad Prism 6.0 and a parametric unpaired t‐test was performed where, ***p < 0.001, NS = not significant. Ectopic (E) TBX2 and (F) TBX3 protein levels detected by western blotting using a FLAG antibody in inducible TBX2‐FLAG 501mel and inducible TBX3‐FLAG 501mel cells respectively induced with doxycycline (dox) for 24 h followed by 10 μM drug (2.5 μM for pyrvinium pamoate due to cell sensitivity) or vehicle treatment for 24 h.

The selected ‘hit’ drugs were next re‐tested in the individual FLAG‐TBX2 and FLAG‐TBX3 inducible 501mel cell lines, and all except pyrvinium pamoate successfully inhibited FLAG‐TBX2 and FLAG‐TBX3 expression (Figure 4). Vardenafil hydrochloride, niclosamide and tacrolimus significantly downregulated (*p* < 0.001) FLAG‐TBX2 (Figure 4B,E), and piroctone olamine and tacrolimus significantly downregulated (*p* < 0.001) FLAG‐TBX3 (Figure 4D,F). Interestingly, in the validation experiments, 10 μM of pyrvinium pamoate almost completely killed the cells. We therefore repeated the assay with 2.5 μM of this drug which did not result in a significant decrease of FLAG‐TBX3 (Figure 4C,D).

**FIGURE 5:**
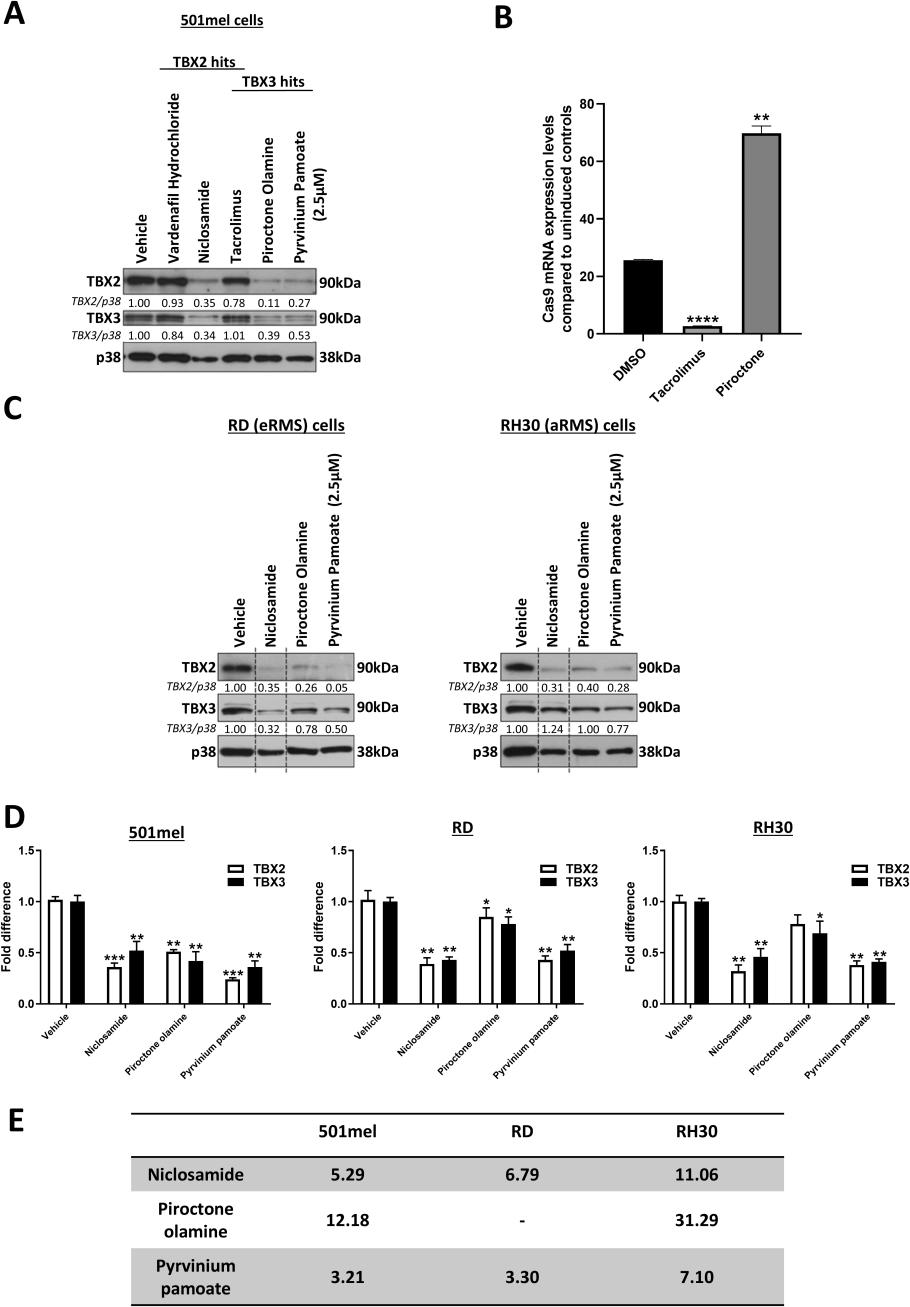
Niclosamide, piroctone olamine and pyrvinium pamoate regulation of TBX2 and TBX3 and the effect of tacrolimus on another Tet‐On inducible system. (A) Endogenous TBX2 and TBX3 levels in 501mel parental cell lines following 24 h treatment with 10 μM drug (2.5 μM for pyrvinium pamoate due to cell sensitivity) or vehicle determined by western blotting using antibodies to TBX2 and TBX3. (B) Quantitative RT‐PCR performed on reverse transcribed RNA extracted from Tet‐On inducible Cas9 stem cells induced with 20 ng/mL doxycycline (dox) for 24 h followed by treatment with 10 μM tacrolimus, piroctone olamine and vehicle for 24 h. Primers specific to Cas9 were used, mRNA levels were normalised to β‐actin and expressed relative to the uninduced control. (C) Western blotting showing levels of TBX2 and TBX3 in ERMS (RD cell line) and ARMS (RH30 cell line) cells after 24 h of 10 μM drug (2.5 μM pyrvinium pamoate) or vehicle treatment. (D) Quantitative RT‐PCR performed on reverse transcribed RNA extracted from 501mel, RD and RH30 cells treated for 24 h treatment with 10 μM drug (2.5 μM for pyrvinium pamoate due to cell sensitivity) or vehicle. Primers specific to TBX2 and TBX3 were used, mRNA levels were normalised to GUSB and expressed relative to the vehicle control. Data was analysed using GraphPad Prism 6.0 and a parametric unpaired t‐test was performed where, *p < 0.05, **p < 0.01, ***p < 0.001 an ****p < 0.0001. (E) Table showing the EC_50_ values from MTT cell viability assays of 501mel, RD and RH30 cells treated with a range of selected ‘Hit’ drug concentrations and vehicle as indicated for 72 h.

We next tested the effect of 10 μM of niclosamide, vardenafil hydrochloride, piroctone olamine and tacrolimus and 2.5 μM pyrvinium pamoate on endogenous TBX2 and TBX3 levels in parental 501mel cells (Figure 5A). While endogenous TBX2 protein levels were minimally affected by vardenafil hydrochloride and tacrolimus, they were effectively downregulated by niclosamide. Piroctone olamine and pyrvinium pamoate effectively downregulated endogenous TBX3 but, as was the case for TBX2, tacrolimus had no effect on TBX3 levels. Unexpectedly, the TBX3 ‘hit’ drugs piroctone olamine and pyrvinium pamoate, were also able to inhibit endogenous TBX2 levels and the TBX2 ‘hit’ drug niclosamide, was able to inhibit endogenous TBX3 levels. A possible explanation for this is that these drugs were identified as ‘hits’ for their ability to downregulate ectopic TBX2/3, but they may be able to directly and or indirectly transcriptionally downregulate endogenous TBX2/3.

We hypothesised that the failure to reproduce the inhibitory effect of tacrolimus and vardenafil hydrochloride on endogenous TBX2/3 levels could be due to these ‘hit’ drugs interfering with the Tet‐On system that drives ectopic TBX2/3 transcription and not through directly inhibiting TBX2/3 levels. To test this, induced pluripotent stem cells (iPSCs) engineered to express Cas9 using the Tet‐On system, were induced and treated with 10 μM tacrolimus or 10 μM piroctone olamine (included as a negative control) or vehicle and Cas9 mRNA levels were measured. As hypothesised, tacrolimus but not piroctone olamine, reduced Cas9 levels (Figure 5B). It is thus possible that tacrolimus and vardenafil hydrochloride prevent doxycycline from activating the Tet‐On system and consequently the expression of FLAG‐TBX2/3.

We also hypothesised that the ‘hit’ drugs may inhibit endogenous TBX2 and/or TBX3 in other TBX2/3‐driven cancers including rhabdomyosarcoma (RMS). To test this, two RMS cell lines RH30 and RD, which represent the alveolar RMS (ARMS) and the embryonal RMS (ERMS) subtypes respectively, were treated with niclosamide, piroctone olamine, pyrvinium pamoate or vehicle and levels of TBX2 and TBX3 were assessed. Figure 5C shows that all three drugs robustly downregulated TBX2 in the RH30 cells and both TBX2 and TBX3 in the RD cells. Interestingly, of the 3 drugs tested, only pyrvinium pamoate decreased TBX3 levels in the RH30 cell line. Together these findings suggested that niclosamide, piroctone olamine and pyrvinium pamoate inhibit TBX2 levels in TBX2‐driven RH30 and RD cells and inhibit TBX3 levels in the TBX3‐driven RD cells. This is an important finding as it shows that these three drugs maintain their ability to downregulate either TBX2, TBX3 or both in TBX2/TBX3‐dependent non‐melanoma cancer cell lines.

Niclosamide, piroctone olamine, and pyrvinium pamoate have been shown to exhibit anti‐cancer activity through their ability to inhibit signalling pathways such as the WNT/B‐catenin that can transcriptionally activate TBX2/TBX3 [28, 29, 30]. We were therefore interested to investigate the effect of these drugs on TBX2/TBX3 mRNA levels. Our data show that niclosamide, piroctone olamine, and pyrvinium pamoate treatment resulted in a significant decrease in the levels of TBX2 and TBX3 mRNA, which suggested that the three drugs are also able to inhibit TBX2 and TBX3 by targeting their upstream activators (Figure 5D).

### 3.5 Niclosamide, Piroctone Olamine and Pyrvinium Pamoate Inhibit Melanoma and RMS Cell Viability

To investigate the anti‐cancer activity of niclosamide, piroctone olamine and pyrvinium pamoate in melanoma and RMS cells, their EC_50_ values were calculated where possible. As shown in Figure 5E, the 501mel, RD and RH30 cells were most sensitive to pyrvinium pamoate with EC_50_ values of 3.21 μM, 3.30 μM and 7.10 μM respectively, followed by niclosamide with EC_50_ values of 5.29 μM, 6.79 μM and 11.06 μM respectively. In the cell lines tested, piroctone olamine had the least effect with EC_50_ values of 12.18 μM in the 501mel and 31.29 μM in the RH30 cell lines, and it was not possible to calculate an EC_50_ value for the RD cell line.

**FIGURE 6:**
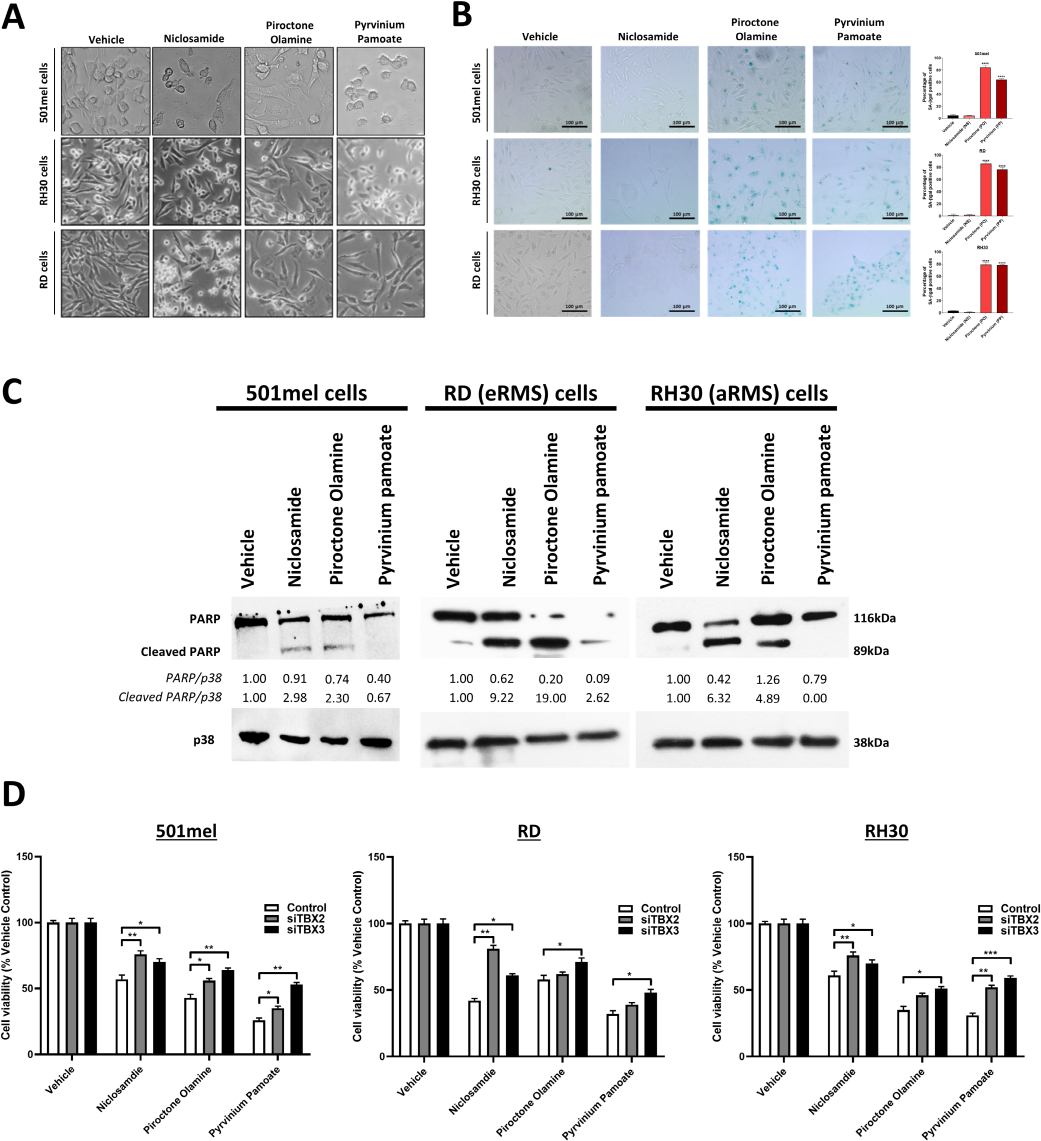
The effect of selected ‘hit’ drugs on TBX2 and TBX3 levels in melanoma, ERMS and ARMS cell lines. (A) Representative light microscopy images (200X; EVOS XL AMEX1000 Core Imaging System) of 501mel, RH30 and RD after 24 h of 10 μM drug (2.5 μM pyrvinium pamoate) or vehicle treatment. (B) Senescence‐Associated (SA)‐β‐Galactosidase staining of 501mel, RD and RH30 cells treated with either vehicle, or EC_50_ niclosamide, piroctone olamine and pyrvinium pamoate for 72 h after which cells were incubated with the SA‐β‐Gal chromogenic substrate 5‐bromo‐4‐chloro‐3‐indolyl β‐D‐galactopyranoside (X‐Gal) at pH 6 and SA‐β‐Gal positive cells were identified by a distinct blue colour. Representative brightfield microscopy images (200X; EVOS M5000 Imaging System; scale bars = 100 μm) are shown. (C) Western blot analyses of protein harvested from 501mel, RD and RH30 cells treated vehicle or EC_50_ concentrations of niclosamide, piroctone olamine and pyrvinium pamoate for 72 h and incubated with the PARP specific antibody. (D) 501mel, RD and RH30 cells were pre‐treated with 20 nM siControl, siTBX2 or siTBX3 24 h prior to the addition of EC_50_ concentrations of niclosamide, piroctone olamine and pyrvinium pamoate for 48 h after which the MTT cell viability assay was performed. p38 was used as a loading control and densitometry readings were obtained using ImageJ and protein expression levels are represented as a ratio of protein of interest/β‐actin normalised to the vehicle control sample (where possible). Blots are representative of at least two independent repeats. Graphs show mean cell viability as a percentage of vehicle control ± SEM for each concentration of drug determined from three independent experiments performed in quadruplicate. Data was analysed using GraphPad Prism 6.0 and a parametric unpaired t‐test was performed where, *p < 0.05, **p < 0.01, ***p < 0.001, NS = not significant.

Light microscopy images of cells treated with the ‘hit’ drugs show that niclosamide, piroctone olamine and pyrvinium pamoate treatment reduced cell densities and induced morphological changes characteristic of senescence and/or apoptosis. Indeed, cells treated with piroctone olamine and pyrvinium pamoate become more cobblestoned, flatter and had enlarged nuclei which are typical characteristics of senescent cells (Figure 6A). To confirm this, 501mel, RD and RH30 cells were treated with the 3 ‘hit’ drugs and senescence‐associated β‐galactosidase (SA‐β‐Gal) activity was measured. Briefly, the drug treated cells were incubated with the SA‐β‐Gal chromogenic substrate 5‐bromo‐4‐chloro‐3‐indolyl β‐D‐galactopyranoside (X‐Gal) at pH 6 and SA‐β‐Gal positive cells were identified by a distinct blue colour. The results show that, compared to vehicle treated cells, while niclosamide did not induce senescence, there was a significantly higher number of SA‐β‐gal positive 501mel, RD and RH30 cells when treated with piroctone olamine or pyrvinium pamoate (Figure 6B). These results are consistent with TBX2 and TBX3 promoting senescence bypass in melanoma cells as previously shown [31].

As indicated above, the light microscopy images of the cells treated with the ‘hit’ drugs also displayed apoptotic characteristics which included cell shrinkage and rounding (Figure 6A). To confirm if the drugs were eliciting cytotoxicity through apoptosis, western blotting was performed with lysates from cells treated with the drugs and an antibody to PARP which detects its cleaved product which is a well‐characterised marker of apoptosis. The data show that niclosamide and piroctone olamine significantly increased the levels of cleaved PARP in 501mel, RD and RH30 cells and pyrvinium pamoate was only able to induce PARP cleavage in the RD cell line (Figure 6C).

We next determined whether the 3 ‘hit’ drugs could inhibit melanoma and rhabdomyosarcoma cell viability through negatively regulating TBX2/3 levels. Briefly, 501mel, RD and RH30 cells were treated with vehicle or drug and then transiently transfected with small interfering RNA specific to TBX2 (siTBX2) or TBX3 (siTBX3) or a control siRNA (siControl), after which MTT cell viability assays were performed. As expected, treatment with niclosamide, piroctone olamine and pyrvinium pamoate significantly reduced the viability of all three cell lines (Figure 6D). Importantly, in 501mel cells, the depletion of TBX2 or TBX3 significantly reduced the cytotoxic effects of niclosamide, piroctone olamine and pyrvinium pamoate. Furthermore, reduced levels of TBX2 or TBX3 in the RD cell line significantly reduced the efficacy of niclosamide but only silencing TBX3 reduced the effects of piroctone olamine and pyrvinium pamoate on cell viability. Lastly, when TBX2 or TBX3 were depleted in the RH30 cells, niclosamide and pyrvinium pamoate were both less effective and once more the cytotoxic effect of piroctone olamine was dependent on the levels of TBX3 (Figure 6D). This data suggest that the cytotoxicity displayed by niclosamide, piroctone olamine and pyrvinium pamoate is in part dependent on the levels of TBX2/TBX3 and that they also exert cytotoxicity through their ability to inhibit key oncogenic pathways such as the WNT/β‐catenin, STAT3 and NOTCH pathways.

## 4 Discussion

Cancer presents a major therapeutic challenge and amongst the promising strategy for developing new therapeutics is the targeting of key molecular factors that drive the oncogenic phenotypes. In this regard, the transcription factors TBX2 and TBX3 are of particular interest because their overexpression in several carcinomas and sarcomas was shown to enhance cell proliferation, bypass senescence and apoptosis, and promote tumour formation, angiogenesis and metastasis [9, 10]. Importantly, they have been biologically validated as potential drug targets in several cancer types including melanoma, breast cancer and rhabdomyosarcoma [11, 32, 33]. However, the drug discovery and development pipeline are extremely arduous, expensive, and risky and therefore repurposing of non‐cancer drugs for anti‐cancer therapy has become a popular cost and time effective alternative [34, 35]. This study therefore adopted a novel strategy by combining a targeted approach with drug repurposing and identified and validated niclosamide, piroctone olamine, and pyrvinium pamoate as ‘hits’. Indeed, these ‘hit’ drugs were shown to display potent cytotoxicity in melanoma and RMS cells and therefore have the potential to be repurposed for the treatment of TBX2/3‐driven cancers.

While the notion of drug repurposing as a rapid, cost‐effective approach to identifying anti‐cancer therapies is still a fledgling field, there are already exciting success stories such as Raloxifene, originally prescribed for osteoporosis and now repurposed for breast cancer, and Aspirin which is used to treat analgesia and repurposed to combat colorectal cancer. Although niclosamide, piroctone olamine and pyrvinium pamoate are not currently approved to treat cancer, there is a growing body of evidence that they exhibit anti‐cancer properties, and this study provides new data to support this. Niclosamide is an FDA‐approved anthelmintic that has historically been used to treat parasitic infections, such as tapeworms and it is also used as a molluscicide in the control of schistosomiasis. In vitro studies have explored niclosamide for the treatment of several cancers including breast, colon, ovarian, prostate, lung, glioblastoma and osteosarcoma where it can inhibit cell migration, invasion, proliferation and induce apoptosis [36, 37, 38, 39, 40, 41, 42]. In addition, in vivo studies show that oral administration of niclosamide reduced tumour growth in mouse models of colon, ovarian, adrenocortical carcinomas as well as glioblastoma with no observed toxicity [28, 29, 37, 41]. Mechanistically, niclosamide was shown to disrupt mitochondrial function and inhibit key oncogenic pathways such as WNT/β‐catenin, STAT3, NOTCH and mTOR [30, 43]. Pyrvinium pamoate is also an FDA‐approved anthelmintic used to treat enterobiasis caused by *Enterobius vermicularis* (pinworm) [43, 44, 45]. It has also been reported to display anti‐cancer activity in colon, pancreatic, breast, bladder, and prostate cancer as well as some haematological malignancies including lymphoma and myeloma and recently, it was found to have potent anti‐breast cancer stem cell activity. Importantly, intraperitoneal administration of pyrvinium pamoate has been efficacious against several of these cancers [46, 47, 48, 49, 50, 51]. Furthermore, pyrvinium pamoate was shown to exert anti‐cancer activity through autophagy depletion, and inhibition of Akt, Wnt/β‐catenin and PI3K‐dependent pathways [50, 52, 53]. Lastly, piroctone olamine is an antifungal agent found in anti‐dandruff shampoos used to treat seborrheic dermatitis and dry scalp [54]. While not as extensively studied as the others, piroctone olamine has been shown to significantly decrease myeloma and lymphoma cell viability by inducing apoptosis and to be a potent WNT/β‐catenin pathway inhibitor in a panel of cancers such as myeloma, lymphoma, and renal and pancreatic cancer [55, 56, 57]. Furthermore, combination therapy of daily oral piroctone olamine and the currently used myeloma therapeutic lenalidomide was shown to have a significant additive effect in an in vivo myeloma model. Importantly, no side effects were observed for piroctone olamine [58, 59].

While the existing safety profiles of the anthelminthics niclosamide and pyrvinium pamoate support their potential fast‐tracking for repurposing as anti‐cancer agents, they have pharmacokinetic limitations, particularly in terms of absorption and systemic bioavailability. Indeed, niclosamide exhibits low systemic bioavailability (approximately 10%) due to its poor water solubility (approximately 5–8 μg/mL at 20°C) which while beneficial for treating local parasitic infections of the intestines is not desirable for the treatment of cancer [60, 61]. There are only a few bioavailability studies for pyrvinium pamoate and the data are variable [62]. For example, while there were no detectable levels of pyrvinium pamoate in the blood plasma or urine samples of patients in which the drug was given orally, it was detected in the blood plasma at a concentration of around 90 nM in mice who received the drug orally [44, 63]. Piroctone olamine is currently used for the topical treatment of dandruff and while it is absorbed through the skin the systemic effects are negligible [64]. To the best of our knowledge, there is only a single study that investigated the effect of orally administered piroctone olamine in a murine myeloma model and the authors reported that there were no detrimental side effects [59]. Together, these studies reveal that repurposing niclosamide, pyrvinium pamoate and piroctone olamine as anti‐cancer agents requires further carcinogenic studies in animal models including exploring alternative drug delivery methods such as administering them intravenously, intrathecally, subcutaneously or intraperitoneally.

This study shows that niclosamide, piroctone olamine and pyrvinium pamoate downregulate TBX2 and TBX3 in 501mel melanoma cells and RD ERMS cells and downregulate TBX2 in RH30 ARMS cells with negligible effects on TBX3 levels. Understanding the mechanism(s) by which these drugs exert their anti‐cancer activity may provide insight into how they are downregulating TBX2/3. This is important because it will shed light on versatile ways of targeting TBX2 and TBX3 in cancer therapies. In this regard, it is worth noting that there is evidence to suggest that niclosamide, piroctone olamine, and pyrvinium pamoate selectively target several oncogenic pathways with the Wnt/β‐catenin pathway being a common target. It is tempting to speculate that the ability of these drugs to inhibit TBX2 and TBX3 may be through their inhibition of the Wnt signalling pathway because it has been previously reported to regulate TBX2 in pancreatic and prostate cancer and TBX3 in liver cancer. Indeed, Chen et al. (2008) showed that the inhibition of β‐catenin in pancreatic cancer cells reduced the expression of TBX2 and Nandana et al. (2017) showed that Wnt signalling is an essential mediator of TBX2 in prostate cancer metastasis and that blocking the pathway through neutralising antibodies or a Wnt antagonist blocked TBX2‐induced invasion in prostate cancer cells [65, 66]. On the other hand, Renard et al. (2007) identified TBX3 as a downstream target gene activated by the Wnt/β‐catenin signalling pathway in liver cancer. Importantly, the authors show that siRNA mediated inhibition of TBX3 impaired β‐catenin‐mediated cell survival and rendered cells sensitive to doxorubicin‐induced apoptosis [67]. Future studies will therefore have to investigate if the Wnt/β‐catenin pathway is involved and, if so, how it achieves this downregulation of TBX2/3. Taken together, it would thus appear that there is an intricate link between the Wnt/β‐catenin pathway and TBX2/3 and that any drug that inhibits this pathway may negatively impact TBX2/3‐driven cancers.

In addition to the Wnt/β‐catenin pathway, niclosamide, piroctone olamine and pyrvinium pamoate have also been reported to target other oncogenic pathways. For example, niclosamide inhibits Notch, NF‐κB and JAK/STAT and pyrvinium pamoate can target Hedgehog, Hippo and PI3K‐dependant pathways [63, 68]. While there is no evidence that the Hedgehog, Hippo, Notch, NF‐κB and JAK/STAT pathways are regulators of TBX2/3, the PI3K pathway has been shown to regulate both TBX2 and TBX3 in melanoma cells [26, 69]. Indeed, TBX3 has been shown to be regulated by AKT3 and AKT1 (key mediators of PI3K signalling) through posttranslational phosphorylation resulting in stabilisation and nuclear localisation in melanoma and fibrosarcoma cells respectively [69, 70]. This suggests that pyrvinium pamoate may potentially be downregulating TBX2 and TBX3 through the PI3K pathway. Furthermore, it will be worth investigating the effect of the Hippo, Hedgehog, Notch, NF‐κB and JAK/STAT pathways on TBX2/3 because it may unravel a network of oncogenic pathways that converge on TBX2/3 and provide more versatile ways of inhibiting their oncogenic functions.

Although the high‐throughput screen was conducted successfully with the identification of ‘hit’ drugs that could be validated, there were several technical challenges that should be noted for the improvement of similar such experiments in the future. For example, as a positive control for the screen, we needed an agent known to negatively regulate TBX2/3 levels and nuclear localisation. Several compounds have been reported to inhibit TBX2/3 levels and or nuclear localisation including the PI3K inhibitors LY294002 and GDC0941 and the nucleolin aptamer AS1411 [26, 71]. These compounds were tested, but none of them provided a large enough dynamic range for the robust detection of ‘hit’ drugs, and thus the experiment proceeded without positive controls. While this is not uncommon, it was risky, and it is highly recommended for more confident identification of ‘hits’ that future screens utilise positive controls. In this regard, the current screen has revealed niclosamide, piroctone olamine and pyrvinium pamoate as potential positive controls. Although the use of the Tet‐On system to express inducible FLAG‐tagged TBX2/3 proteins allowed for better detection of TBX2/3 levels by immunofluorescence with an antibody to FLAG, this system was flawed. For example, only ‘hits’ that post‐transcriptionally regulate TBX2/3 could be identified, and it presented with the risk of identifying false positive ‘hits’ such as tacrolimus that interfere with the Tet‐On system. Therefore, the use of the Tet‐On system or similar systems in such a drug screen should only be used if a better alternative is not available.

In summary, this study adopted a target‐based drug repurposing strategy to identify commercial drugs that negatively regulate the oncogenic TBX2 and TBX3 transcription factors. While several challenges were encountered in designing and developing the screen, the screen was successfully run, and ‘hits’ were identified and selected ‘hits’ were validated. This study shows for the first time that niclosamide, piroctone olamine and pyrvinium pamoate can negatively regulate the oncogenic TBX2 and TBX3, and it also broadens the anti‐cancer activity of these drugs to include efficacy against melanoma and rhabdomyosarcoma. Although our findings are promising, further validation and characterisation are required to confirm the potential of repurposing niclosamide, piroctone olamine and pyrvinium pamoate for the treatment of, at the very least, TBX2/3‐driven cancers.

## Author Contributions

**Jenna S. Bleloch:** data curation (equal), formal analysis (equal), investigation (equal), methodology (equal), validation (equal), visualization (equal), writing – original draft (equal). **Sizhu Lu:** conceptualization (equal), data curation (equal), formal analysis (equal), investigation (equal), validation (equal). **Saif Feroz Khan:** investigation (equal), validation (equal), writing – review and editing (equal). **Karabo Serala:** investigation (equal), validation (equal), writing – review and editing (equal). **Daniel Ebner:** formal analysis (equal), investigation (equal), methodology (equal), resources (equal), supervision (equal), visualization (equal). **Colin Goding:** conceptualization (equal), formal analysis (equal), funding acquisition (equal), resources (equal), supervision (equal), writing – review and editing (equal). **Sharon Prince:** conceptualization (equal), formal analysis (equal), funding acquisition (equal), resources (equal), supervision (equal), writing – original draft (equal), writing – review and editing (equal). **Val Millar:** formal analysis (equal), methodology (equal). **Elena Seraia:** formal analysis (equal), methodology (equal).

## Conflicts of Interest

The authors declare no conflicts of interest.

## Data Availability

The data generated in this study are available within the article and are available from the corresponding author upon reasonable request.

## References

[1] S. Ciavarella , A. Milano , F. Dammacco , and F. Silvestris , “Targeted Therapies in Cancer,” BioDrugs 24 (2010): 77–88, 10.2165/11530830-000000000-00000. 20199123

[2] S. J. Johnston and J. S. Carroll , “Transcription Factors and Chromatin Proteins as Therapeutic Targets in Cancer,” Biochimica et Biophysica Acta (BBA)–Reviews on Cancer 1855 (2015): 183–192, 10.1016/j.bbcan.2015.02.002. 25721328

[3] V. E. Papaioannou , “The T‐Box Gene Family: Emerging Roles in Development, Stem Cells and Cancer,” Development 141 (2014): 3819–3833, 10.1242/dev.104471. 25294936 PMC4197708

[4] R. J. R. J. Bollag , Z. Siegfried , J. A. J. A. Cebra‐Thomas , N. Garvey , E. M. E. M. Davison , and L. M. L. M. Silver , “An Ancient Family of Embryonically Expressed Mouse Genes Sharing a Conserved Protein Motif With the T Locus,” Nature Genetics 7 (1994): 383–389, 10.1038/ng0794-383. 7920656

[5] A. Sebé‐Pedrós , A. Ariza‐Cosano , M. T. Weirauch , et al., “Early Evolution of the T‐Box Transcription Factor Family,” Proceedings of the National Academy of Sciences 110 (2013): 16050–16055, 10.1073/pnas.1309748110. PMC3791752 24043797

[6] M. Bamshad , T. Le , W. S. Watkins , et al., “The Spectrum of Mutations in TBX3: Genotype/Phenotype Relationship in Ulnar‐Mammary Syndrome,” American Journal of Human Genetics 64 (1999): 1550–1562. 10330342 10.1086/302417 PMC1377898

[7] C. Campbell , K. Goodrich , G. Casey , and B. Beatty , “Cloning and Mapping of a Human Gene (TBX2) Sharing a Highly Conserved Protein Motif With the Drosophila Omb Gene,” Genomics 28 (1995): 255–260, 10.1006/geno.1995.1139. 8530034

[8] D. J. Law , T. Gebuhr , N. Garvey , S. I. Agulnik , and L. M. Silver , “Identification, Characterization, and Localization to Chromosome 17q21‐22 of the Human TBX2 Homolog, Member of a Conserved Developmental Gene Family,” Mammalian Genome 6, no. 11 (1995): 793–797. 8597636 10.1007/BF00539006

[9] S. F. S. F. Khan , V. Damerell , R. Omar , et al., “The Roles and Regulation of TBX3 in Development and Disease,” Gene 726 (2020): 144223, 10.1016/j.gene.2019.144223. 31669645 PMC7108957

[10] A. Abrahams , M. I. Parker , and S. Prince , “The T‐Box Transcription Factor Tbx2: Its Role in Development and Possible Implication in Cancer,” IUBMB Life 62 (2010): 92–102, 10.1002/iub.275. 19960541

[11] S. Wansleben , J. Peres , S. Hare , C. R. Goding , and S. Prince , “T‐Box Transcription Factors in Cancer Biology,” Biochimica et Biophysica Acta (BBA)–Reviews on Cancer 1846 (2014): 380–391, 10.1016/j.bbcan.2014.08.004. 25149433

[12] T. Willmer , A. Cooper , D. Sims , D. Govender , and S. Prince , “The T‐Box Transcription Factor 3 Is a Promising Biomarker and a Key Regulator of the Oncogenic Phenotype of a Diverse Range of Sarcoma Subtypes,” Oncogene 5 (2016): e199, 10.1038/oncsis.2016.11. PMC5154352 26900951

[13] B. Zhu and J. K. Davie , “New Insights Into Signalling‐Pathway Alterations in Rhabdomyosarcoma,” British Journal of Cancer 112 (2015): 227–231, 10.1038/bjc.2014.471. 25211658 PMC4453439

[14] J. Peres and S. Prince , “The T‐Box Transcription Factor, TBX3, Is Sufficient to Promote Melanoma Formation and Invasion,” Molecular Cancer 12 (2013): 117, 10.1186/1476-4598-12-117. 24098938 PMC3851469

[15] S. Prince , S. Carreira , K. W. Vance , A. Abrahams , and C. R. Goding , “Tbx2 Directly Represses the Expression of the P21(WAF1) Cyclin‐Dependent Kinase Inhibitor,” Cancer Research 64 (2004): 1669–1674. 14996726 10.1158/0008-5472.can-03-3286

[16] E. Davis , H. Teng , B. Bilican , et al., “Ectopic Tbx2 Expression Results in Polyploidy and Cisplatin Resistance,” Oncogene 27 (2008): 976–984. 17700536 10.1038/sj.onc.1210701

[17] S. Pushpakom , F. Iorio , P. A. Eyers , et al., “Drug Repurposing: Progress, Challenges and Recommendations,” Nature Reviews. Drug Discovery 18 (2019): 41–58, 10.1038/nrd.2018.168. 30310233

[18] T. T. Ashburn and K. B. Thor , “Drug Repositioning: Identifying and Developing New Uses for Existing Drugs,” Nature Reviews. Drug Discovery 3 (2004): 673–683, 10.1038/nrd1468. 15286734

[19] P. N. Aguiar , J. J. Adashek , F. Roitberg , C. M. Noia Barreto , A. del Giglio , and G. L. Lopes , “In the Era of Cost‐Effectiveness Analysis, Affordability Is a Limiting Factor for Patients' Access to Innovative Cancer Treatments,” Value in Health Regional Issues 20 (2019): 47–50, 10.1016/j.vhri.2018.12.003. 30856543

[20] J. Schmidt , Role and Regulation of TBX2 and TBX3 (University of Oxford, 2015).

[21] A. N. Goktug , S. C. Chai , and T. Chen , “Data Analysis Approaches in High Throughput Screening,” in Drug Discovery (InTech, 2013), 10.5772/52508.

[22] X. D. Zhang , “Illustration of SSMD, *z* Score, SSMD*, Z* Score, and t Statistic for Hit Selection in RNAi High‐Throughput Screens,” Journal of Biomolecular Screening 16 (2011): 775–785, 10.1177/1087057111405851. 21515799

[23] S. Prince , T. Wiggins , P. A. Hulley , and S. H. Kidson , “Stimulation of Melanogenesis by Tetradecanoylphorbol 13‐Acetate (TPA) in Mouse Melanocytes and Neural Crest Cells,” Pigment Cell Research 16 (2003): 26–34, 10.1034/j.1600-0749.2003.00008.x. 12519122

[24] C. A. Schneider , W. S. Rasband , and K. W. Eliceiri , “NIH Image to ImageJ: 25 Years of Image Analysis,” Nature Methods 9 (2012): 671–675, 10.1038/nmeth.2089. 22930834 PMC5554542

[25] J. S. Bleloch , A. du Toit , L. Gibhard , et al., “The Palladacycle Complex AJ‐5 Induces Apoptotic Cell Death While Reducing Autophagic Flux in Rhabdomyosarcoma Cells,” Cell Death Discovery 5 (2019): 60, 10.1038/s41420-019-0139-9. 30701092 PMC6349869

[26] S. Lu , P. Louphrasitthiphol , N. Goradia , et al., “TBX2 Controls a Proproliferative Gene Expression Program in Melanoma,” Genes & Development 35 (2021): 1657–1677, 10.1101/gad.348746.121. 34819350 PMC8653791

[27] M. Rodriguez , E. Aladowicz , L. Lanfrancone , and C. R. Goding , “Tbx3 Represses E‐Cadherin Expression and Enhances Melanoma Invasiveness,” Cancer Research 68 (2008): 7872–7881, 10.1158/0008-5472.CAN-08-0301. 18829543

[28] M. L. King , M. E. Lindberg , G. R. Stodden , et al., “WNT7A/β‐Catenin Signaling Induces FGF1 and Influences Sensitivity to Niclosamide in Ovarian Cancer,” Oncogene 34 (2015): 3452–3462, 10.1038/onc.2014.277. 25174399 PMC4345161

[29] K. Satoh , L. Zhang , Y. Zhang , et al., “Identification of Niclosamide as a Novel Anticancer Agent for Adrenocortical Carcinoma,” Clinical Cancer Research 22 (2016): 3458–3466, 10.1158/1078-0432.CCR-15-2256. 26873959 PMC4947455

[30] Bayer Pharmaceuticals Corporation , YOMESAN® [Package Insert] (Connecticut, USA: West Haven, 1988).

[31] K. W. Vance , S. Carreira , G. Brosch , and C. R. Goding , “Tbx2 Is Overexpressed and Plays an Important Role in Maintaining Proliferation and Suppression of Senescence in Melanomas,” Cancer Research 65 (2005): 2260–2268, 10.1158/0008-5472.CAN-04-3045. 15781639

[32] B. Zhu , M. Zhang , S. D. Byrum , A. J. Tackett , and J. K. Davie , “TBX2 Blocks Myogenesis and Promotes Proliferation in Rhabdomyosarcoma Cells,” International Journal of Cancer 135 (2014): 785–797, 10.1002/ijc.28721. 24470334 PMC4051830

[33] D. A. Sims , “The Role of T‐Box Transcription Factor TBX3 in Rhabdomyosarcoma,” 2016.

[34] F. Pammolli , L. Magazzini , and M. Riccaboni , “The Productivity Crisis in Pharmaceutical R&D,” Nature Reviews. Drug Discovery 10 (2011): 428–438, 10.1038/nrd3405. 21629293

[35] M. J. Waring , J. Arrowsmith , A. R. Leach , et al., “An Analysis of the Attrition of Drug Candidates From Four Major Pharmaceutical Companies,” Nature Reviews. Drug Discovery 14 (2015): 475–486, 10.1038/nrd4609. 26091267

[36] W. Lu , C. Lin , M. J. Roberts , W. R. Waud , G. A. Piazza , and Y. Li , “Niclosamide Suppresses Cancer Cell Growth by Inducing Wnt co‐Receptor LRP6 Degradation and Inhibiting the Wnt/β‐Catenin Pathway,” PLoS One 6 (2011): 1–8, 10.1371/journal.pone.0029290. PMC3241710 22195040

[37] T. Osada , M. Chen , X. Y. Y. Yang , et al., “Antihelminth Compound Niclosamide Downregulates Wnt Signaling and Elicits Antitumor Responses in Tumors With Activating APC Mutations,” Cancer Research 71 (2011): 4172–4182, 10.1158/0008-5472.CAN-10-3978. 21531761 PMC3117125

[38] R. C. Arend , A. I. Londoño‐Joshi , R. S. Samant , et al., “Inhibition of Wnt/β‐Catenin Pathway by Niclosamide: A Therapeutic Target for Ovarian Cancer,” Gynecologic Oncology 134 (2014): 112–120, 10.1016/j.ygyno.2014.04.005. 24736023

[39] C. Liu , W. Lou , Y. Zhu , et al., “Niclosamide Inhibits Androgen Receptor Variants Expression and Overcomes Enzalutamide Resistance in Castration‐Resistant Prostate Cancer,” Clinical Cancer Research 20 (2014): 3198–3210, 10.1158/1078-0432.CCR-13-3296. 24740322 PMC4058390

[40] R. Li , Z. Hu , S.‐Y. S. Y. Sun , et al., “Niclosamide Overcomes Acquired Resistance to Erlotinib Through Suppression of STAT3 in Non‐Small Cell Lung Cancer,” Molecular Cancer Therapeutics 12 (2013): 2200–2212, 10.1158/1535-7163.MCT-13-0095. 23894143 PMC3795834

[41] A. Wieland , D. Trageser , S. Gogolok , et al., “Anticancer Effects of Niclosamide in Human Glioblastoma,” Clinical Cancer Research 19 (2013): 4124–4136, 10.1158/1078-0432.CCR-12-2895. 23908450

[42] M. Chen , J. Wang , J. Lu , et al., “The Anti‐Helminthic Niclosamide Inhibits Wnt/Frizzled1 Signaling,” Biochemistry 48 (2009): 10267–10274, 10.1021/bi9009677. 19772353 PMC2801776

[43] FDA , Approved Drug Products With Therapeutic Equivalence Evaluations, 38th ed. (Silver Spring, USA: U.S. Department of Health and Human Services, 2018).

[44] T. C. Smith , A. W. Kinkel , C. M. Gryczko , and J. R. Goulet , “Absorption of Pyrvinium Pamoate,” Clinical Pharmacology and Therapeutics 19 (1976): 802–806. 1269218 10.1002/cpt1976196802

[45] J. A. Turner and P. E. Johnson , “Pyrvinium Pamoate in the Treatment of Pinworm Infection (Enterobiasis) in the Bome,” Journal of Pediatrics 60 (1962): 243–251, 10.1016/S0022-3476(62)80042-0. 13923208

[46] H. Esumi , J. Lu , Y. Kurashima , and T. Hanaoka , “Antitumor Activity of Pyrvinium Pamoate, 6‐(Dimethylamino)‐2‐[2‐(2,5‐Dimethyl‐1‐Phenyl‐1H‐Pyrrol‐3‐Yl)Ethenyl]‐1‐Methyl‐Quinolinium Pamoate Salt, Showing Preferential Cytotoxicity During Glucose Starvation,” Cancer Science 95 (2004): 685–690, 10.1111/j.1349-7006.2004.tb03330.x. 15298733 PMC11159109

[47] B. Li , D. L. Fei , C. A. Flaveny , et al., “Pyrvinium Attenuates Hedgehog Signaling Downstream of Smoothened,” Cancer Research 74 (2014): 4811–4821, 10.1158/0008-5472.CAN-14-0317. 24994715 PMC4321822

[48] D.‐H. Yu , J. Macdonald , G. Liu , et al., “Pyrvinium Targets the Unfolded Protein Response to Hypoglycemia and Its Anti‐Tumor Activity Is Enhanced by Combination Therapy,” PLoS One 3 (2008): e3951, 10.1371/journal.pone.0003951. 19079611 PMC2597738

[49] D. Basu , M. Reyes‐Múgica , and A. Rebbaa , “Histone Acetylation‐Mediated Regulation of the Hippo Pathway,” PLoS One 8 (2013): e62478, 10.1371/journal.pone.0062478. 23671600 PMC3646011

[50] Y. Harada , I. Ishii , K. Hatake , et al., “Pyrvinium Pamoate Inhibits Proliferation of Myeloma/Erythroleukemia Cells by Suppressing Mitochondrial Respiratory Complex I and STAT3,” Cancer Letters 319 (2012): 83–88, 10.1016/J.CANLET.2011.12.034. 22210382

[51] J. Guo , J. Lv , S. Chang , et al., “Inhibiting Cytoplasmic Accumulation of HuR Synergizes Genotoxic Agents in Urothelial Carcinoma of the Bladder,” Oncotarget 7 (2016): 45249–45262, 10.18632/oncotarget.9932. 27303922 PMC5216720

[52] A. A. Momtazi‐borojeni , E. Abdollahi , F. Ghasemi , M. Caraglia , and A. Sahebkar , “The Novel Role of Pyrvinium in Cancer Therapy,” Journal of Cellular Physiology 233 (2017): 2871–2881. 28500633 10.1002/jcp.26006

[53] A. Wiegering , F. W. Uthe , M. Hüttenrauch , et al., “The Impact of Pyrvinium Pamoate on Colon Cancer Cell Viability,” International Journal of Colorectal Disease 29 (2014): 1189–1198, 10.1007/s00384-014-1975-y. 25060218

[54] P. Klug and R. Kreiling , Safety and Effectiveness Information for Piroctone Olamine (Germany: Frankfurt/Main, 2004).

[55] I. Wall and I. G. H. Schmidt‐Wolf , “Effect of Wnt Inhibitors in Pancreatic Cancer,” Anticancer Research 34 (2014): 5375–5380. 25275031

[56] C. M. Koller , Y. Kim , and I. G. H. Schmidt‐Wolf , “Targeting Renal Cancer With a Combination of WNT Inhibitors and a bi‐Functional Peptide,” Anticancer Research 33 (2013): 2435–2440. 23749892

[57] S. A. Von Schulz‐Hausmann , L. C. Schmeel , F. C. Schmeel , and I. G. H. Schmidt‐Wolf , “Targeting the Wnt/Beta‐Catenin Pathway in Renal Cell Carcinoma,” Anticancer Research 34 (2014): 4101–4108. 25075035

[58] S. Song , T. Christova , S. Perusini , et al., “Wnt Inhibitor Screen Reveals Iron Dependence of ‐Catenin Signaling in Cancers,” Cancer Research 71 (2011): 7628–7639, 10.1158/0008-5472.CAN-11-2745. 22009536

[59] Y. Kim , P. Alpmann , S. Blaum‐Feder , et al., “Increased In Vivo Efficacy of Lenalidomide by Addition of Piroctone Olamine,” In Vivo 25 (2011): 99–103. 21282741

[60] M. O. Jara and R. O. Williams, III , “The Challenge of Repurposing Niclosamide: Considering Pharmacokinetic Parameters, Routes of Administration, and Drug Metabolism,” Journal of Drug Delivery Science and Technology 81 (2023): 104187, 10.1016/j.jddst.2023.104187.

[61] B. Devarakonda , R. A. Hill , W. Liebenberg , M. Brits , and M. M. de Villiers , “Comparison of the Aqueous Solubilization of Practically Insoluble Niclosamide by Polyamidoamine (PAMAM) Dendrimers and Cyclodextrins,” International Journal of Pharmaceutics 304 (2005): 193–209, 10.1016/j.ijpharm.2005.07.023. 16198076

[62] F. M. Ponzini , C. W. Schultz , B. E. Leiby , et al., “Repurposing the FDA‐Approved Anthelmintic Pyrvinium Pamoate for Pancreatic Cancer Treatment: Study Protocol for a Phase I Clinical Trial in Early‐Stage Pancreatic Ductal Adenocarcinoma,” BMJ Open 13 (2023): e073839, 10.1136/bmjopen-2023-073839. PMC10582846 37848297

[63] C. W. Schultz and A. Nevler , “Pyrvinium Pamoate: Past, Present, and Future as an Anti‐Cancer Drug,” Biomedicine 10 (2022): 3249, 10.3390/biomedicines10123249. PMC9775650 36552005

[64] C. F. Tang , M. Paz‐Alvarez , P. D. A. Pudney , and M. E. Lane , “Characterization of Piroctone Olamine for Topical Delivery to the Skin,” International Journal of Cosmetic Science 45 (2023): 345–353, 10.1111/ics.12839. 36752028 PMC10947268

[65] S. Nandana , M. Tripathi , P. Duan , et al., “Bone Metastasis of Prostate Cancer Can be Therapeutically Targeted at the TBX2–WNT Signaling Axis,” Cancer Research 77 (2017): 1331–1344, 10.1158/0008-5472.CAN-16-0497. 28108510 PMC5783646

[66] P. Chen , D. Tian , and M. Liu , “The Role of Tbx2 in Pancreatic Cancers and Its Regulation by Wnt/β‐Catenin Signaling,” Chinese‐German Journal of Clinical Oncology 7 (2008): 404–409, 10.1007/s10330-008-0054-7.

[67] C. Renard , C. Labalette , C. Armengol , et al., “Tbx3 Is a Downstream Target of the Wnt/Beta‐Catenin Pathway and a Critical Mediator of Beta‐Catenin Survival Functions in Liver Cancer,” Cancer Research 67 (2007): 901–910. 17283120 10.1158/0008-5472.CAN-06-2344

[68] H. Jiang , A. M. Li , and J. Ye , “The Magic Bullet: Niclosamide,” Frontiers in Oncology 12 (2022): 12, 10.3389/fonc.2022.1004978. PMC9720275 36479072

[69] J. Peres , S. Mowla , and S. Prince , “The T‐Box Transcription Factor, TBX3, Is a Key Substrate of AKT3 in Melanomagenesis,” Oncotarget 6 (2015): 1821–1833, 10.18632/oncotarget.2782. 25595898 PMC4359334

[70] R. Omar , A. Cooper , H. M. Maranyane , et al., “COL1A2 is a TBX3 target that mediates its impact on fibrosarcoma and chondrosarcoma cell migration,” Cancer Letters 459 (2019), 10.1016/j.canlet.2019.06.004. 31202624

[71] T. Willmer , V. Damerell , S. Smyly , et al., “Targeting the Oncogenic TBX3:Nucleolin Complex to Treat Multiple Sarcoma Subtypes,” American Journal of Cancer Research 11 (2021): 5680–5700. 34873487 PMC8640805

